# Vehicle-to-Pedestrian Communication for Vulnerable Road Users: Survey, Design Considerations, and Challenges

**DOI:** 10.3390/s19020358

**Published:** 2019-01-17

**Authors:** Parag Sewalkar, Jochen Seitz

**Affiliations:** Communication Networks Group, Technical University of Ilmenau, 98693 Ilmenau, Germany; Jochen.Seitz@tu-ilmenau.de

**Keywords:** V2X, V2P, Vulnerable Road Users, Vehicle-to-Pedestrian, 802.11p

## Abstract

In the last few years, increasing attention has been provided to research Vehicle-to-Pedestrian (V2P) communication systems. These V2P systems serve different purposes (safety or convenience) and cater to different Vulnerable Road User (VRU) groups. Also, these V2P systems employ different communication technologies, and use different mechanisms to interact with the users. An effective V2P system also needs to consider varying characteristics of different VRUs. These various elements may be considered as design parameters of the V2P system. In this paper, we discuss such elements and propose a design framework for the V2P system based on them. We also provide an extensive survey of existing V2P efforts for safety and convenience applications and their design considerations. We perform a case study that compares the different approaches of V2P safety system for different VRU groups under different pre-crash scenarios. Finally, we discuss a few technological challenges in integration of VRUs into V2X systems.

## 1. Introduction

**Figure 1:**
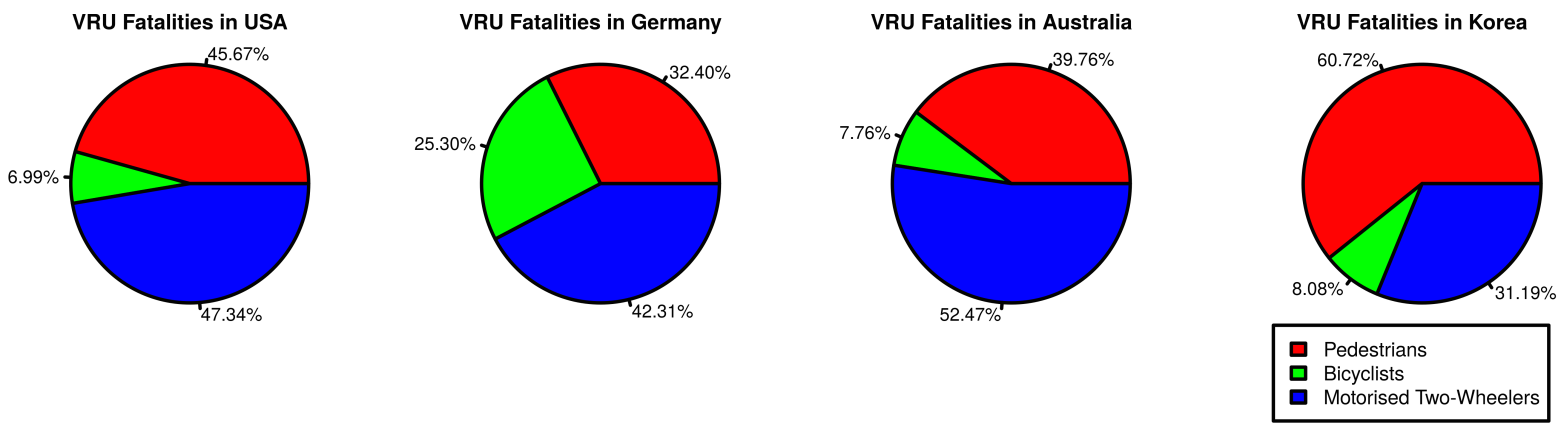
VRU Fatalities by VRU Type.

Pedestrians, cyclists, and motorized two-wheeler operators are called Vulnerable Road Users (VRUs). According to the International Traffic Safety Data and Analysis Group (IRTAD), in 2012, there were 1605 and 10,386 VRU fatalities in Germany and USA respectively [1]. Figure 1 shows the proportion of different types of VRU fatalities in the USA, Germany, Australia, and Korea [1]. It shows that VRU groups have differing rates of fatalities among different countries. There have been numerous advancements to improve safety features of vehicles as part of Intelligent Transportation Systems (ITS). These safety features help improve safety of vehicle-occupants as well as VRUs. Vehicle-to-Everything (V2X) communication is one such safety feature that establishes communication among various entities on road for co-operative safety. V2X involves communication between Vehicle-to-Vehicle (V2V), Vehicle-to-Infrastructure (V2I), and Vehicle-to-Pedestrian (V2P). V2P is an umbrella term that encompasses the communication between vehicles and all types of VRUs. By enabling V2P for VRUs, they can become active part of ITS and can enable various safety and convenience ITS applications.

VRUs differ in their characteristics, such as, speed, mobility, travel patterns. For example, pedestrians travel slowly compared to cyclists and motorized two-wheelers. Another example is that motorized two-wheelers must stop at intersection during red light but pedestrians may cross the road in the same duration. V2P system developers must consider these varying characteristics for designing an effective V2P system. The characteristics may be translated into appropriate design requirements for the V2P system. The clearly defined requirements then may help address different challenges in VRU integration. The requirements may fall under various categories, e.g., type of VRU, the pre-crash scenario. We discuss each of these categories in later part of this paper. There have been multiple efforts to solve different problems in integration of VRUs into ITS [2,3,4,5,6,7,8,9]. Also, multiple real-life pilot projects, such as, VRUITS, InDev, XCYCLE, PROSPECT, have been undertaken in order to identify, understand, and address the VRU needs [10,11,12,13]. These efforts deploy various mechanisms to meet one or more criteria tailored to the targeted VRU group. We discuss the mechanisms and challenges that are presented by these efforts. Also, there have been no efforts to evaluate the various V2X approaches and pre-crash scenarios. We perform a case study of prominent communication mechanisms in the context of various crash scenarios and assess their feasibility.

This paper is organized as follows. Section 2 gives a brief overview of basic V2P architecture. In Section 3, we discuss various characteristics of V2P systems further classifying them and discussing their impact on V2P systems. In Section 4, we present our case study concept, simulations and evaluation of results. In Section 5, we discuss important aspects of the V2P systems. Section 6 focuses on open research issues and possible future directions.

## 2. V2P System Architecture

A typical V2P crash prevention system involves periodic exchange of safety messages among vehicles and VRUs. This communication can happen either directly using ad-hoc communication technologies, such as IEEE 802.11p, or indirectly using infrastructure-based communication, such as, cellular technology. Also, V2P system performs its operation in three phases: detection, tracking and trajectory prediction, and action [14]. These elements have led to different V2P system architectures. In this section, we briefly discuss the different V2P architectural components and the safety messages.

### 2.1. Components

V2P systems can be broadly classified into the following components:

1. Vehicle device
2. VRU device
3. Infrastructure
4. Information processing unit

**Figure 2:**
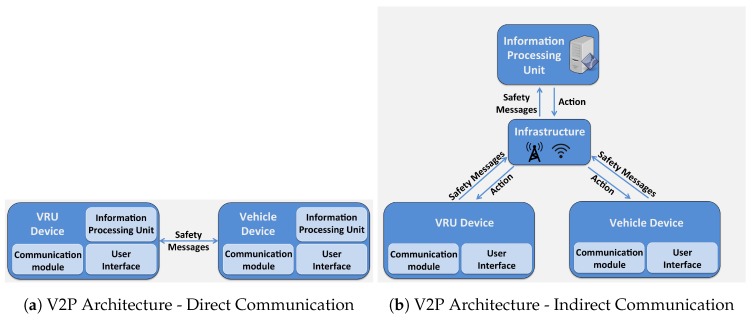
Example V2P System Architectures.

If V2P system relies on direct communication then the system comprises of only two components viz. *Vehicle device* and *VRU device*. These two components are responsible to carry all three phases of the V2P system. Efforts by [3,4,15] are examples of such system. However, if the V2P system relies on indirect communication (i.e., through infrastructure) then an *Information processing unit* is responsible to carry out *detection, tracking and trajectory prediction* phases. It determines the possibility of a crash based on the trajectory prediction. It then notifies the *Vehicle device* and *VRU device* through *Infrastructure* for necessary action, if required. *Vehicle device* and *VRU device* may then carry out the necessary *action* phase. Efforts by [5,16] are examples of such a system. Figure 2 depicts examples of different V2P system architectures.

### 2.2. Safety Messages

A typical safety message may contain speed, location, and direction of the respective vehicle or VRU. This information may then be used for the *detection, tracking and trajectory prediction* phases by the recipients of the safety message. Vehicles may transmit 10 safety messages per second (i.e., at fixed 10 Hz frequency). VRUs may transmit safety messages with varying frequency. This frequency may depend on various parameters, such as, their location and speed.

## 3. Classification

We broadly classify the design inputs of VRU integration into total 8 categories. These categories are discussed in this section.

### 3.1. Types of VRUs

VRUs groups, i.e., pedestrians, cyclists, and motorized two-wheeler operators vary in their characteristics and pre-crash scenarios. In this subsection, we discuss the characteristics of different types of VRUs and various efforts targeting specific types of VRUs.

#### 3.1.1. Pedestrians

Pedestrians’ typical walking speed is 1.4 m/s (5 km/h). Pedestrians may walk alone or in groups of various sizes. Walking speed of pedestrians may vary by age and physical ability. Based on pedestrians’ physical characteristics, they may further be classified into following groups:

1. Adults—This group adheres to the typical characteristics of pedestrians, such as speed and trajectory.
2. Children—This group may exhibit characteristics such as unpredictable trajectory, slow walking or running.
3. Senior and physically disadvantaged persons—This group may exhibit characteristics such as slow walking and may use some assistance (e.g., cane, wheelchair, or a guide dog).

Multiple efforts have been made to research and design V2P crash prevention systems for aforementioned pedestrian groups [3,4,5,9,15,17,18,19,20,21,22,23,24,25,26,27,28,29,30,31]. These efforts use various approaches to achieve their goal of V2P system. Table 1 lists various V2P systems with their key features. Here we discuss a few approaches.

Wu et al. [3] have designed a DSRC-based V2P system for pedestrians. It uses a smartphone as a VRU device and leverages the smartphone sensors in order to optimize the transmission of safety messages. Both VRU device and vehicle, send safety messages that contain their location, speed, and direction information. Anaya et al. [4] propose V2ProVu, a Wi-Fi-based system, that alerts pedestrians for potential crash. It uses a smartphone as a VRU device. This system requires a VRU device to listen for safety messages sent by vehicles and then predict the collision probability. Sugimoto et al. [5] propose a V2P system that enables the exchange of safety messages through a combination of cellular infrastructure and direct Wi-Fi communication. A central information processing server processes the safety messages that it receives from vehicles and pedestrians and calculates the collision risk. Dhondge et al. [15] propose WiFiHonk, a Wi-Fi-based system, that enables vehicles and pedestrians to exchange safety messages without camping on Wi-Fi network. The system stuffs Wi-Fi beacons with safety messages in order to achieve this goal. Lewandowski et al. [21] propose an IEEE 802.15.4 based V2P system that involves a *Warning Unit* as a vehicle equipment and a *Tag* as a VRU device. The system is based on a paging process where the *Warning Unit* sends “Hello” messages to which the *Tag* responds by sending “Here I am” packet. The tag can be put into children’s backpacks in order to make vehicles aware of the children’s presence. The Ko-TAG project [27] proposes a co-operative pedestrian localization system. This system requires the pedestrian to carry a transponder that communicates with the vehicle’s on-board localization unit. This communication helps vehicles to localize the pedestrians and bicyclists.

#### 3.1.2. Cyclists

Cyclists’ usual traveling speed is 4.2 m/s (15 km/h). Cyclists travel on the road alone. Even when in groups, they may travel in a line following each other. Multiple efforts have been made to design V2P crash prevention systems for cyclists [17,22,27,35,36,38,39,40].

C-AEB [36] have designed an ITS-G5-based system that enables vehicles and cyclists to exchange safety messages. In this system, the vehicle tracks the cyclist based on the received safety messages. It then fuses the data from safety messages with the data it receives from other sensors, such as Radar and camera. This approach helps improve reliability of all sensors. Thielen et al. [37] propose a heterogeneous and infrastructure-assisted approach to establish communication between vehicle and cyclist. The cyclist’s device (smartphone) sends safety messages to the Road-Side Unit (RSU) using Wi-Fi. The RSU relays this information to the nearby vehicle using ITS-G5. Anaya et al. [39] propose “MotoWarn”, a system that uses Bluetooth and iBeacon technology to inform vehicles about the cyclists’ presence. The vehicle is equipped with a V2X device that also has a Bluetooth interface. This Bluetooth interface can receive the iBeacon messages sent by cyclists.

#### 3.1.3. Motorized Two-Wheelers (MTW)

Motorized two-wheelers’ typical traveling speed, in urban area, is 14 m/s (50 km/h) and they are the fastest group of VRUs. MTWs usually travel on the road alone. A few efforts have been made to design V2P crash prevention systems for motorized two-wheelers [8,39].

“MotoWarn” [39] supports a system that informs the vehicle of the motorcycle’s presence in real-time using 802.11p. The system equips both, the vehicle and the motorcycle, with a V2X-capable unit and establishes a unidirectional communication (from motorcycle to vehicle). Based on the information received from the motorcycle, the vehicle system then predicts the collision probability and warns the driver, if necessary. Huang et al. [8] propose the “RedEye” system that helps prevent collisions caused by scooters that violate red-light. RedEye uses the scooter rider’s smartphone to detect the red-light violation. It then warns the rider to slow down and also warns the nearby vehicles. RedEye also receives warnings sent by other RedEye-enabled riders.

### 3.2. Pre-Crash Scenarios

**Figure 3:**
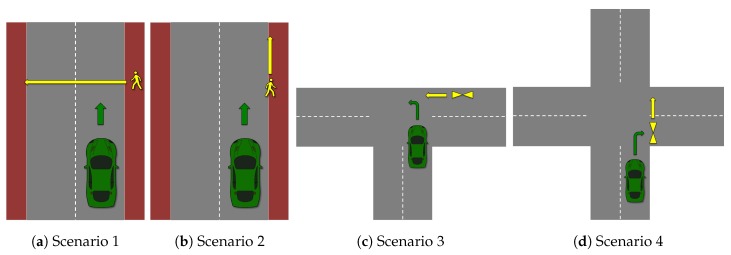
Various pre-crash scenarios. (**a**) Pedestrian crossing in front of vehicle; (**b**) Pedestrian moving parallel to vehicle; (**c**) Vehicle turning left into cyclist’s path (**d**) Vehicle turning right into cyclist’s path.

Pre-crash scenarios may help understand the requirements of an effective V2P crash prevention system. The pre-crash scenarios for different types of VRUs may differ from each other. Pedestrian fatality analysis shows that 88% of the pedestrian fatalities are tied to a scenario where a pedestrian is crossing the road in front of a vehicle moving on a straight road and 12% are tied to a scenario where the pedestrian is moving on a straight road parallel to a vehicle’s direction [43]. Also, the majority of the pedestrian crashes occur at non-junctions [44]. In case of cyclists, the majority of crashes happen when a vehicle is turning right or left into the cyclist’s path [45]. Also, the majority of cyclist crashes occur at intersections or junctions [45]. Figure 3 depicts the most common pre-crash scenarios for pedestrians and cyclists. In these scenarios, various factors may cause the crash. A few examples of such factors are obstructed view, speed of the vehicle and VRU, visibility (day/night) etc.

V2P system developers may design crash prevention systems that are adapted to these pre-crash scenarios in order to maximize effectiveness.

### 3.3. Mode of Communication

V2P systems for VRUs may achieve the communication among vehicles and VRUs through direct or indirect communication. Furthermore, there are hybrid modes, too.

#### 3.3.1. Direct

*Direct* mode of communication involves vehicles and VRUs communicating with each other *directly* i.e., without any intermediate entity. References [3,4,15,21,28,39] are some examples of such direct mode that use various technologies for communication. This may be the fastest mode of communication among all three modes due to its ability to establish direct communication. This mode may be best suited for safety applications due to lower latency in communication. However, it requires that all devices be equipped with same type of communication technology. This may pose deployment challenges. It also requires devices to process the received safety messages locally which may require high computing power. Also, due to its direct nature, the range of communication is limited by the underlying technology.

#### 3.3.2. Indirect

*Indirect* mode of communication involves vehicles and VRUs communicating with each other *indirectly* i.e., through infrastructure. References [7,20,32,37,38,41] are a few examples of the V2P systems for VRUs that use the indirect mode of communication. The devices may be equipped with same technology, such as cellular [20], or with different technologies, such as 802.11p and Wi-Fi [37]. As the exchange of messages happens through the infrastructure, the system may first process the messages in infrastructure nodes before forwarding them to other nodes. This may require the infrastructure nodes to have high computing power. Also, the exchange of messages through infrastructure nodes may cause higher communication latency. This imposes the requirement that the latency constraint of the target V2P application must be assessed against the infrastructure latency.

A variation of the *Indirect* mode may be *multi-hop* communication. In case of Non-Line-of-Sight (NLOS) crash scenarios, multi-hop communication may be useful. A vehicle may re-broadcast the safety message that it receives from a VRU to the surrounding vehicles. For example, a public transport bus, at the bus-stop, may re-broadcast the safety messages from the VRUs crossing the street in front of the bus.

#### 3.3.3. Hybrid

*Hybrid* mode of communication involves vehicles and VRUs communicating with each other *directly* using ad-hoc communication as well as *indirectly* through infrastructure. This may be achieved by equipping the devices with multiple communication technologies and designing the system that may leverage the capabilities of these technologies. References [5,25] are a few examples of the V2P systems for VRUs that use the hybrid mode of communication. This mode may overcome the limitations of *direct* and *indirect* modes i.e., communication range and communication latency respectively. However, this mode increases the system complexity as it requires the precise partitioning of the functionalities of various communication technologies in the system.

### 3.4. Type of Applications

Based on the type of application, V2P systems for VRUs may be classified broadly into two categories, namely *safety* and *convenience*.

#### 3.4.1. Safety Applications

Safety applications are the V2P crash prevention systems. There may be multiple V2P safety applications that may each address different types of VRU, pre-crash scenarios, and vehicles. Table 1 lists various V2P safety efforts that target different types of VRUs. There have also been efforts to deploy applications for specific groups of pedestrians and vehicles [46,47]. *Mobile Accessible Pedestrian Signal System* is an application deployed for visually impaired pedestrians for signalized street crossing scenario [46]. *Pedestrian in Signalized Crosswalk Warning* is a V2P safety application that warns public transport bus operators about the pedestrians that are in the path of the bus at signalized intersection [47].

#### 3.4.2. Convenience Applications

Convenience applications are the V2P applications that assist the VRUs by improving their travel efficiency through various services. Examples of such convenience applications are ride-sharing, green light for bicycles, traffic information for VRUs etc. Tal et al. [40] propose a V2P-based convenience application that helps electric bicycles save battery power. This is achieved in real-time by calculating the bike’s recommended speed based on the received traffic light timer information. The TIMON project [42] provides various convenience services for VRUs that include current traffic status, information about vehicle collisions, and re-routing assistance. Table 1 lists various V2P convenience applications.

### 3.5. Notification Recipients

As discussed in Section 3.4.1, there can be multiple V2P safety applications addressing different aspects of crash preventions (type of VRU, scenario etc.). Also, V2P safety application capabilities are also dependent on the type of VRU device and the underlying communication technology. These factors may lead V2P systems to have different recipients of crash warnings.

1. Driver: In this V2P system, when a vehicle-VRU crash is predicted, only the vehicle’s driver is notified so that further action may be taken. References [21,27,28] are examples of such systems. Notification to the driver may be a symbol on vehicle’s dashboard.
2. VRU: In this V2P system, when a vehicle-VRU crash is predicted, only the VRU is notified. Reference [4,15,38] are examples of such system. Notification to the VRU may be in form of audio-visual warning on the VRU device.
3. Both: In this V2P system, when a vehicle-VRU crash is predicted, both nodes (vehicle and VRU) are notified. References [3,5,26] are the examples of such a system.

Table 1 shows various efforts by their notification recipient.

Similarly, notification recipients for the V2P convenience applications may also be classified into above three categories. However, we are not aware of enough number of efforts for V2P convenience applications to draw any conclusion.

### 3.6. Communication Technologies

As Table 1 shows, various communication technologies have been used to design V2P systems. Some characteristics of the V2P systems largely depend upon the choice of the underlying communication technology. Examples of such characteristics are the range of communication, the choice of V2P device, the availability of infrastructure etc. In this section, we briefly discuss each communication technology and its characteristics.

#### 3.6.1. 802.11p

IEEE 802.11p, which operates in 5.9 GHz frequency, has been specifically designed for V2X communications. It can support the exchange of safety messages reliably and with low latency even under the typical high vehicular mobility conditions. However, it requires VRU devices to be equipped with 802.11p. Although Reference [3] has shown the feasibility of equipping smartphone with 802.11p, we are not aware of any smartphone or other VRU device that supports 802.11p off-the-shelf. This may pose deployment problems. 802.11p based systems typically support a communication range up to 1 km which may be enough even at high speeds e.g., 40 m/s. 802.11p-based systems may be deployed with infrastructure [37,39] or without [3,26,36]. Due to its reliability and low latency, 802.11p technology is a good candidate for V2P safety applications.

#### 3.6.2. Cellular

A few efforts [5,25,38] have been made to design V2P safety systems using cellular technology. All of the efforts use 3G or Long Term Evolution (LTE) for communication and smartphones as a VRU device. Cellular-based V2P systems typically have a longer communication range due to the use of central infrastructure. However, latency and scalability performance of cellular V2P systems need to be researched further in order to determine their suitability for V2P safety systems. Due to its widespread coverage and high market penetration, cellular system is a good candidate for V2P convenience applications.

Cellular V2X (C-V2X) is currently under development as part of the proposed 5G architecture. Once fully developed, C-V2X promises to fulfill the requirements of various use cases of V2X communications including V2P [48]. However, we are not aware of any efforts that have used C-V2X for design or evaluation of a V2P system.

#### 3.6.3. Wi-Fi

Multiple efforts [4,8,9,15,23,24,35] have been made to design Wi-Fi-based V2P safety systems. These systems use a smartphone as a VRU device and typically have 100–150 m of communication range. This range may be enough in urban areas with typical vehicle speeds up to 50 km/h. However, it may not be enough in suburban areas with typical speeds of 100 km/h due to less time available for the driver’s reaction to crash warnings. Also, Wi-Fi’s association requirement is a challenge due to the mobility of vehicles as it may take too much time before the actual exchange of safety messages happens. Wi-Fi-based V2P systems may be deployed without the help of infrastructure.

#### 3.6.4. Localization

Schaffer et al. [27] have developed a co-operative pedestrian localization system that can operate at 2.44 GHz and 5.768 GHz. It uses a special tag as a VRU device which can directly communicate with the vehicle’s device without any infrastructure. This V2P system achieves a communication range up to 100 m. Scalability and latency performance of this system need to be researched further due to its requirement of fixed tag identification numbers.

#### 3.6.5. Bluetooth

Anaya et al. [39] have developed a Bluetooth-based V2P safety system for bicyclists. This system uses iBeacon as a VRU device in order to communicate with the vehicle directly. It achieves a communication range of up to 50 m which may be enough for a particular pre-crash scenario e.g., Figure 3d. However, due to its limited communication range, Bluetooth may not be able to support V2P in its entirety. For example, it may support only urban scenarios with slower speeds.

#### 3.6.6. 700 MHz ITS Band

Nagai et al. [28] have developed a system for V2P communication in 700 MHz ITS band (in accordance to the Japanese standard for ITS). It evaluates various channel access mechanisms for V2P system. It serves as a proof-of-concept for co-existence of V2P, V2V and V2I systems in the 700 MHz system.

#### 3.6.7. 802.15.4

Lewandowski et al. [21] is an example of the system based on 802.15.4 that we have discussed in Section 3.1.1. 802.15.4 technology can achieve the communication range of up to 80 m. 802.15.4-based systems may be helpful where only unilateral notification of the collision (to the vehicle’s driver) is sufficient.

### 3.7. VRU Devices

Various pre-crash scenarios and varying capabilities of VRUs impose the constraint of functionality and accessibility on VRU devices. For example, smartphones may be good devices for adults but not children. In this section, we discuss various options that may be used as VRU devices.

#### 3.7.1. Smartphone

Due to their versatility and ubiquitous nature, smartphones may prove themselves as most widely accepted choice as a VRU device. It can also be seen from Table 1 that 23 out of 28 systems for V2P communication have used a smartphone as a VRU device. Current commercial off-the-shelf (COTS) smartphones already pack the various sensors, such as, accelerometer, GPS, and communication technologies, such as, cellular (LTE/3G), Bluetooth, Wi-Fi etc. By fusing functionalities of these sensors and communication technologies, effective V2P systems may be developed. Smartphones can also provide the necessary functionality for audio-visual and haptic warnings. This may be useful for V2P systems that incorporate warnings for VRUs.

#### 3.7.2. Helmet

Helmets may be used as a VRU device for cyclists and MTWs. However, this requires that the helmet be equipped with the necessary components that enable it to be used as a VRU device. Hernandez-Jayo et al. [38] use a helmet and smartphone as a VRU device for the cyclist. In this system, the smartphone is used to transmit the position data to the cloud and the helmet is used to warn the cyclist about the presence of the vehicle.

#### 3.7.3. Tag

A tag may be used as a VRU device in the V2P systems where unilateral warning (only to vehicle driver) is necessary or sufficient. It may be placed in the children’s backpack, wheelchairs, handbags, etc. [21,27] use a tag as a VRU device. The tag may not participate in V2P communication actively and may reply only when the vehicle device is detected.

### 3.8. Role of VRU Devices

The mechanism how VRU devices participate in V2P communication may be categorized in two categories, namely, *active* and *passive*. Table 1 shows the existing efforts that are also classified by the VRU device role.

#### 3.8.1. Active

When a VRU device participates actively in V2P communication, by sending information about the VRU location, speed, etc. periodically, it may be called as active participation. This requires the VRU device to be equipped with multiple technologies, such as, GPS, communication technologies. This type of participation is more widely used as seen from Table 1. This type of participation may increase network congestion caused by VRU safety messages adversely impacting potentially more crucial V2V and V2I communication [14]. Hence this type of system may need to employ mechanisms to optimize VRU transmissions.

#### 3.8.2. Passive

When the VRU device only ’listens’ to the messages from a vehicle and/or when VRU device sends a reply only when it detects the message from a vehicle, this type of participation may be called as passive participation. In a V2P system where the VRU device only listens to the messages from vehicles, vehicles may not be aware of the VRU’s presence. This requires the VRUs to be aware of vehicle to avoid potential crashes. Also, in a V2P system where the VRU device replies only when it detects the message from the vehicle, the system completely relies on the reliability and efficiency of the VRU device. Also, performance of such systems in the dense vehicle scenarios remain unseen.

## 4. Case Study of Crash Scenarios

As mentioned in Section 3.2, there are 4 most common pre-crash scenarios for pedestrians and cyclists. To design a crash-prevention system, it is necessary to evaluate various mechanism under these scenarios.

### 4.1. Concept

A good V2P system must be able to effectively operate its three phases, i.e., *detection, tracking and trajectory prediction, and action*. *Detection* phase requires the first contact between a vehicle and VRU [14]. Similarly, *tracking and trajectory prediction* requires sufficient number of messages exchanged, and *action* phase requires sufficient amount of time for reaction in order to stop the vehicle. Our study evaluates the mechanisms of active and passive participation to understand the first contact by VRU, available response time, and number of messages received by the vehicles from the VRU involved in the potential crash.

#### 4.1.1. Active Mechanism

In *Active Mechanism*, VRU devices participate in V2P communication *actively* i.e., they transmit the safety messages periodically. This periodicity may be varied based on the context of the VRU [49]. In our scenario, we use fixed periodicity of 0.5 s.

#### 4.1.2. Passive Mechanism

In *Passive Mechanism*, VRU devices participate in V2P communication *passively* i.e., they transmit the safety messages only when they detect the possibility of a crash. In our scenario, when the VRU device receives the first message from the vehicle that is crashing with the VRU, it waits for two seconds before it transmits the safety message. All subsequent safety messages are then transmitted every two seconds.

### 4.2. Scenario

**Figure 4:**
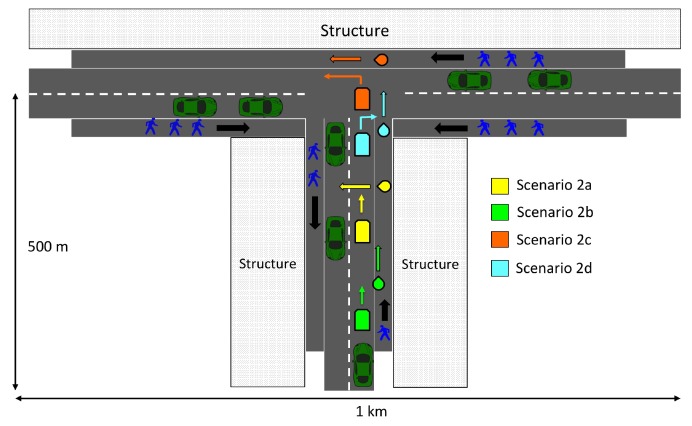
Simulation Scenario.

Our scenario consists of 4 pairs of vehicles and VRUs that correspond to the 4 pre-crash scenarios. This allows us to study the first contact time, number of exchanged messages, and the total contact duration before the crash for each pre-crash scenario. We consider a 802.11p-based V2V and V2P network for our evaluation. Vehicles and pedestrians use 802.11p to exchange safety messages with each other. We consider a T-junction with vehicles and VRU traffic. Figure 4 depicts the scenario with 4 pre-crash configurations. The scenario consists of two roads, with two lanes in either direction, forming a T-Junction and footpaths that are surrounded by building structures. The intersection is controlled by traffic lights. The vehicles, pedestrians, and bicyclists are inserted at the far end of the road/footpath and travel towards intersection. The vehicles’ traveling speed is set to the value that corresponds to urban speed limit.The four pairs of vehicles and VRUs, corresponding to the pre-crash scenarios, travel on the road as shown in Figure 4.

### 4.3. Simulation Environment

We selected OMNeT++, Veins, and Simulation of Urban MObility (SUMO) tools to simulate our V2V/V2P network [50,51,52]. Vehicles are inserted at every 2 s. Pedestrians are inserted at every 1.6 s. Transmission power of Vehicles and VRUs is set to 20 mW. This allows the communication range of 400 m. We employ TwoRayInterferenceModel as a path loss model in order to achieve realistic path propagation of V2P and V2V networks. Table 2 provides the details of simulation parameters used in our scenario.

The four pre-crash scenarios have different warm-up periods and simulation run-times which are shown in Table 3. The warm-up period allows each scenario to get into typical traffic condition. The crash time indicates the simulation time when the vehicle and VRU pair crashes into each other.

The simulation data is collected over 3 independent runs for every pre-crash scenario under active and passive configurations. Average values are then computed for evaluation of final results.

### 4.4. Evaluation

We consider two metrics to evaluate the V2P system under different pre-crash scenarios and mechanisms: Available Response Time for the vehicle and number of safety messages received by the vehicle from the VRU. To calculate the Available Response Time for the vehicle before the crash, we consider the time-stamp of first ever beacon received by the vehicle from the corresponding VRU (*detection* phase). The Available Response Time, for each pre-crash scenario, is give by Equation (1).

(1) ART=CT−FBT

where:

*ART* = Available Response Time
*CT* = Crash Time
*FBT* = First Beacon Time

The number of safety messages, received from the VRU, gives a measure of reliability for *tracking and prediction* phase. We consider the total number of safety messages received by the vehicle, from the corresponding VRU in crash, for this purpose. Table 4 shows the Available Response Time and the number of received safety messages for each pre-crash scenario under *Active* and *Passive* mechanisms.

As expected, the Available Response Time is always less in *Passive* mechanism than in *Active*. This is due to the fact that the VRU device waits for 2 s before the safety message transmission. SAE J2945/9 document indicates that the collision awareness message must be issued 8 s before the crash [49]. Under our simulation scenario, none of the mechanisms can fulfill this condition for scenarios 2.a and 2.c. Scenario 2.a has a short Available Response Time because the VRU has to travel a short distance (<4 m) before it crashes with the vehicle. Scenario 2.c has a short Available Response Time because the VRU and the vehicle are unable to communicate with each other due to NLOS. The scenarios 2.b and 2.d show sufficiently long Available Response Times.

The number of received safety messages are always less in *Passive* mechanism than in *Active*. Also, in scenario 2.a and 2.c, the vehicle receives only 1 safety message from the VRU under *Passive* mechanism. This may only be sufficient for the *detection* phase and no messages are available for *tracking and prediction* phase. Under scenarios 2.b and 2.d, the vehicle receives a sufficiently large number of messages for both the mechanisms.

## 5. Discussion

In this section, we discuss some important aspects of integrating VRUs in V2X communication.

### 5.1. Network Congestion

As pointed out by a few efforts [3,14,49], network congestion can become severe with a large number of VRUs when all VRU devices *actively* participate in the V2P communication. To solve the network congestion issue, a few schemes, such as, Receive-only mode [3], contextual transmission [49], and clustering of VRUs [14] have been suggested.

### 5.2. Location Accuracy

In V2P communication, precise location information is needed to predict the crash probability accurately. However, real-world measurements show that GPS location inaccuracy is 3 m for [3] and 10 m for [4]. This requires crash prediction algorithms to accommodate GPS inaccuracies while calculating the crash probability. Efforts by Audi [17] use a Kalman filter mechanism in order to accommodate GPS inaccuracy for VRU positioning. Broadcom Inc. has developed a positioning solution for a smartphone with 30 cm accuracy [53], however its results in real-world remain unseen.

Also, current GPS-equipped devices (OBU/smartphones) do not support differentiation in 3D plane. This limitation may lead to false positive collision warnings. For example, if a pedestrian is crossing a street using an overpass while a vehicle is passing, the V2P system may predict that the vehicle and the pedestrian are on the verge of collision. The V2P systems must be capable of identifying the situation when the involved entities are not at the same level.

### 5.3. Technology Standardization

As Table 1 shows, various technologies and approaches have been used for V2P systems. These systems vary in their architecture and abilities of communication range, latency, and bandwidth. This makes it difficult to predict the results if these systems were deployed in other scenarios or for other group of VRUs. Hence, it is necessary to standardize the V2P technology keeping various parameters of V2P systems in mind. Also, there is a need to define and standardize the messages that are being exchanged between vehicles and VRUs [17].

### 5.4. Provision of Quality-of-Service (QoS)

When the V2P system predicts that a vehicle-VRU crash is probable, it is necessary to prioritize the communication between the crash-prone pair over other V2P communications. This is required in order to be more certain about the crash so that both the devices (vehicle unit and VRU device) can warn their users about the crash. However, 802.11p-based systems do not provide such a mechanism despite being especially designed for V2X communications [14]. We discuss two possible solutions to address this challenge for 802.11p-based systems.

#### 5.4.1. Request to Lower Priority

The devices of vehicle and VRU, that are on the verge of collision, broadcast a special message requesting other nodes to lower the *priority* of transmission of safety messages while maintaining their own priority. This leads other nodes to lower the priority of their message temporarily (e.g., for next scheduled transmission). This allows the crash-prone devices a higher chance of wireless channel access ensuring the guarantee of message delivery.

#### 5.4.2. Request to Lower Message Periodicity

The devices of vehicle and VRU (that are on the verge of collision) broadcast a special message requesting other nodes to lower the *periodicity* of transmission of safety messages while maintaining their own periodicity. This leads other nodes to lower the periodicity of their message temporarily (e.g., from 10 Hz to 5 Hz for the next scheduled transmission). This allows the crash-prone devices to transmit with higher periodicity ensuring the guarantee of message delivery.

## 6. Open Research Challenges and Future Directions

As integration of VRUs into ITS is being researched, there remain several open research issues. In this section, we discuss these issues and also, possible future directions as follows:

- 3D localization has been widely researched in the research community. However, to the best of our knowledge, there have been no efforts for 3D localization of VRUs in V2P systems.
- Current self-driving vehicle efforts are focused on using standalone technologies, such as, computer vision, Radar, and LiDAR. The results of how the self-driving vehicles respond to V2P-enabled VRU detection remain unseen.
- A V2P-capable vehicle simultaneously need to detect and track (anonymously) multiple VRUs (V2P-capable) that are present in its vicinity. However, current V2P efforts have not fully explored this aspect yet. The number of VRUs that can be detected and tracked (anonymously) simultaneously and the factors that may affect this capability, such as, limitations of object tracking algorithms, are currently unexplored.
- The VRU and vehicles, that are potentially on the verge of collision, may need to communicate with each other. This requires a higher and on-demand QoS in real-time. The algorithms for on-demand QoS for crucial V2P communication, in the presence of rest V2X communication, may pose interesting research problems.
- Mobile Edge Computing (MEC) is currently being researched for V2X networks. MEC may be considered in the design of V2P systems. The role that MEC can perform, in safety as well as convenience V2P applications, is currently unexplored. It may enable V2P safety communication and also, help reduce network congestion caused by VRU-generated safety messages.
- Integration of V2P systems with Geographical Information Systems (GIS) may help enable predictive warnings about VRUs. For example, V2P system may request the information from GIS, such as, school location or bus-stop information, and warn drivers beforehand about VRUs’ presence. GIS may help improve efficiency of V2P systems, including safety as well as convenience, in a specific area.

## 7. Conclusions

V2X systems for VRU safety and convenience are expected to be deployed in coming years. However, it is necessary for V2X systems to incorporate various characteristics of target VRU groups and scenarios. This paper proposes a design framework for the V2P system that may be used to design a system based on the targeted V2P use case. It also provides a survey of existing V2P efforts and identifies their design considerations based on the proposed framework. A detailed discussion is provided about every aspect of the design framework. This paper also performs a comparative case study of the *Active* and *Passive* VRU participation mechanisms under the most prominent pre-crash scenarios for two different VRU groups. The case study shows that the 802.11p-based V2P safety systems must consider additional mechanisms, for some pre-crash scenarios, to provide adequate warnings of eminent collision. The paper also discusses some technological challenges of V2X-VRU integration. In future, we plan to work on the network congestion issue caused by V2X-VRU integration.

## Figures and Tables

**Table 1:** Summary of efforts.

Publication	VRU Type	Mode	Notification Recipient	Type	VRU Device	Technology	Role
Wu et al. [3]	pedestrian	direct	both	safety	smartphone	802.11p	active
V2ProVu [4]	pedestrian	direct	pedestrian	safety	smartphone	Wi-Fi	passive
Sugimoto et al. [5]	pedestrian, cyclist	hybrid	both	safety	smartphone	Cellular, Wi-Fi	active
WiFiHonk [15]	pedestrian	direct	both	safety	smartphone	Wi-Fi	passive
WiSafe [29]	pedestrian	direct	vehicle	safety	smartphone	Wi-Fi	active
Audi [17]	pedestrian, cyclist	direct	both	safety	smartphone	Wi-Fi	active
Lee and Kim [18]	pedestrian	direct	–	safety	smartphone	802.11p	active
David and Flach [19]	pedestrian	hybrid	–	safety	smartphone	Cellular, Wi-Fi	active
Zadeh et al. [30]	pedestrian	indirect	both	safety	smartphone	Cellular	active
pSafety [31]	pedestrian	indirect	both	safety	smartphone	Cellular	active
Artail et al. [32]	pedestrian	indirect	vehicle	safety	smartphone	802.11p, Cellular	passive
Nakanishi et al. [33]	pedestrian	direct	vehicle	safety	smartphone	Wi-Fi	passive
Bagheri et al. [20]	pedestrian	indirect	–	safety	smartphone	Cellular	active
V2PSense [34]	pedestrian	indirect	pedestrian	safety	smartphone	Cellular	passive
LP^3^S [21]	pedestrian	direct	vehicle	safety	tag	802.15.4	passive
General Motors [22]	pedestrian, cyclist	direct	vehicle	safety	smartphone	Wi-Fi	active
Fujikami et al. [9]	pedestrian	direct	-	safety	smartphone	Wi-Fi	active
Liu et al. [23]	pedestrian	direct	both	safety	smartphone	Wi-Fi	active
Hussein et al. [24]	pedestrian	direct	both	safety	smartphone	Wi-Fi	active
Merdrignac et al. [35]	pedestrian	direct	both	safety	smartphone	Wi-Fi	active
POFS [25]	pedestrian	hybrid	pedestrian	safety	smartphone	Cellular, Wi-Fi	active
Tahmasbi-Sarvestani et al. [26]	pedestrian	direct	both	safety	smartphone	802.11p	active
Ko-TAG [27]	pedestrian, cyclist	direct	vehicle	safety	tag	localization	passive
Nagai et al. [28]	pedestrian	direct	vehicle	safety	smartphone	700 MHz ITS	active
C-AEB [36]	cyclist	direct	vehicle	safety	smartphone	802.11p	active
Thielen et al. [37]	cyclist	indirect	vehicle	safety	smartphone	Wi-Fi, 802.11p	active
Hernandez-Jayo et al. [38]	cyclist	indirect	cyclist	safety	helmet, smartphone	Cellular, 802.11p	active
MotoWarn [39]	cyclist	direct	vehicle	safety	iBeacon	Bluetooth	active
MotoWarn [39]	MTW	direct	vehicle	safety	OBU	802.11p	active
RedEye [8]	MTW	direct	both	safety	smartphone	Wi-Fi	active
Tal et al. [40]	cyclist	direct	cyclist	convenience	smartphone	–	passive
Liu et al. [7]	pedestrian	indirect	both	convenience	smartphone	–	active
Lu et al. [41]	pedestrian	indirect	both	convenience	smartphone	802.11p	active
TIMON [42]	cyclist MTW	hybrid	both	convenience	smartphone	Cellular, 802.11p	active

**Table 2:** Simulation Parameters.

Simulation Parameters	Value
Road length	1 km × 500 m
No. of vehicles	120–150
Max. vehicle speed	13.89 m/s = 50 km/h
No. of pedestrians	102
Max. pedestrians speed	1.5 m/s
No. of bicycles	1
Max. bicycle speed	4.3 m/s
Transmission power for vehicles	20 mW
Transmission power for VRUs	20 mW
Data rate	6 Mb/s
Vehicles beacon periodicity	10 Hz
VRU beacon periodicity	2 Hz
(for Active Mechanism)	
Beacon length	1024 bits

**Table 3:** Scenario-specific Parameters.

Scenario	Warm-Up (in s)	Crash Time (in s)	Simulation Length
2.a	30	33.5	33.5
2.b	11	44	44
2.c	45	49	49
2.d	10	48	48

**Table 4:** Results.

Scenario	Available Response Time (before Crash, in s)	Average No. of Received Messages (from VRUs)
2.a		
Active	2.13	5
Passive	0.39	1
2.b		
Active	31.7	62
Passive	29.96	13.33
2.c		
Active	3.65	7.33
Passive	1.27	1
2.d		
Active	37.65	74
Passive	35.93	16.67

## Abbreviations

The following abbreviations are used in this manuscript:

DSRC	Dedicated Short Range Communication
GIS	Geographical Information Systems
ITS	Intelligent Transportation System
MEC	Mobile Edge Computing
MTW	Motorized Two Wheeler
QoS	Quality-of-Service
RSU	Road-Side Unit
VRU	Vulnerable Road User
V2I	Vehicle-to-Infrastructure
V2P	Vehicle-to-Pedestrian
V2V	Vehicle-to-Vehicle
V2X	Vehicle-to-Everything

## References

[1] IRTAD (2014). Road Safety Annual Report 2014 Summary.

[2] Choi P., Goswami S., Radhakrishna U., Khanna D., Boon C.C., Lee H.S., Antoniadis D., Peh L.S. (2015). A 5.9-GHz Fully Integrated GaN Frontend Design With Physics-Based RF Compact Model. IEEE Trans. Microw. Theory Tech..

[3] Wu X., Miucic R., Yang S., Al-Stouhi S., Misener J., Bai S., Chan W. Cars Talk to Phones: A DSRC Based Vehicle-Pedestrian Safety System. Proceedings of the 80th Vehicular Technology Conference (VTC Fall).

[4] Anaya J.J., Merdrignac P., Shagdar O., Nashashibi F., Naranjo J.E. Vehicle to Pedestrian Communications for Protection of Vulnerable Road Users. Proceedings of the Intelligent Vehicles Symposium (IV).

[5] Sugimoto C., Nakamura Y., Hashimoto T. Prototype of pedestrian-to-vehicle communication system for the prevention of pedestrian accidents using both 3G and WLAN communication. Proceedings of the 3rd International Symposium on Wireless Pervasive Computing.

[6] Tang S., Saito K., Obana S. Transmission Control for Reliable Pedestrian-to-Vehicle Communication by Using Context of Pedestrians. Proceedings of the IEEE International Conference on Vehicular Electronics and Safety.

[7] Liu N., Liu M., Cao J., Chen G., Lou W. When Transportation Meets Communication: V2P over VANETs. Proceedings of the 30th International Conference on Distributed Computing Systems (ICDCS).

[8] Huang K.S., Chiu P.J., Tsai H.M., Kuo C.C., Lee H.Y., Wang Y.C.F. (2016). RedEye: Preventing Collisions Caused by Red-Light-Running Scooters With Smartphones. IEEE Trans. Intell. Transp. Syst..

[9] Fujikami S., Sumi T., Yagiu R., Nagai Y. Fast Device Discovery for Vehicle-to-Pedestrian Communication using Wireless LAN. Proceedings of the 12th Annual IEEE Consumer Communications and Networking Conference (CCNC).

[10] VRUITS (2016). VRUITS Project, Improving the Safety and Mobility of Vulnerable Road Users through ITS Applications. https://cordis.europa.eu/project/rcn/186986/factsheet/en.

[11] InDev (2018). InDev Project, In-Depth Understanding of Accident Causation for Vulnerable Road Users. https://www.indev-project.eu.

[12] XCYCLE Project (2018). XCYCLE. http://www.xcycle-h2020.eu/.

[13] Prospect Project, Proactive Safety for Pedestrian and Cyclists (2017). Prospect Project. http://www.prospect-project.eu/.

[14] Sewalkar P., Krug S., Seitz J. Towards 802.11p-based Vehicle-to-Pedestrian Communication for Crash Prevention Systems. Proceedings of the 9th International Congress on Ultra Modern Telecommunications and Control Systems.

[15] Dhondge K., Song S., Choi B.Y., Park H. WiFiHonk: Smartphone-based Beacon Stuffed WiFi Car2X-Communication System for Vulnerable Road User Safety. Proceedings of the IEEE 79th Vehicular Technology Conference.

[16] Bhargava B., Angin P., Duan L. A Mobile-Cloud Pedestrian Crossing Guide for the Blind. Proceedings of the International Conference on Advances in Computing & Communication.

[17] Engel S. Car2Pedestrian: Protection of vulnerable road users using smartphones. Proceedings of the 17th International Forum on Advanced Microsystems for Automotive Applications.

[18] Lee S., Kim D. (2016). An Energy Efficient Vehicle to Pedestrian Communication Method for Safety Applications. Wirel. Pers. Commun..

[19] David K., Flach A. (2010). CAR-2-X and Pedestrian Safety. Veh. Technol. Mag..

[20] Bagheri M., Siekkinen M., Nurminen J.K. (2016). Cloud-Based Pedestrian Road-Safety with Situation- Adaptive Energy-Efficient Communication. IEEE Intell. Transp. Syst. Mag..

[21] Lewandowski A., Boecker S., Koester V., Wietfeld C. Design and Performance Analysis of an IEEE 802.15.4 V2P Pedestrian Protection System. Proceedings of the 5th International Symposium on Wireless Vehicular Communications.

[22] GM Corporate Newsroom (2012). GM Developing Wireless Pedestrian Detection Technology. http://media.gm.com/media/us/en/gm/news.detail.html/content/Pages/news/us/en/2012/Jul/0726_pedestrian.html.

[23] Liu Z., Liu Z., Meng Z., Yang X., Pu L., Zhang L. (2016). Implementation and performance measurement of a V2X communication system for vehicle and pedestrian safety. Int. J. Distrib. Sens. Netw..

[24] Hussein A., Garcia F., Armingol J.M., Olaverri-Monreal C. P2V and V2P Communication for Pedestrian Warning on the basis of Autonomous Vehicles. Proceedings of the IEEE 19th International Conference on Intelligent Transportation Systems (ITSC).

[25] Liu Z., Pu L., Meng Z., Yang X., Zhu K., Zhang L. POFS: A Novel Pedestrian-oriented Forewarning System for Vulnerable Pedestrian Safety. Proceedings of the International Conference on Connected Vehicles and Expo (ICCVE).

[26] Tahmasbi-Sarvestani A., Mahjoub H.N., Fallah Y.P., Moradi-Pari E., Abuchaar O. (2017). Implementation and Evaluation of a Cooperative Vehicle-to-Pedestrian Safety Application. IEEE Intell. Transp. Syst. Mag..

[27] Schaffer B., Kalverkamp G., Chaabane M., Biebl E.M. (2012). A cooperative transponder system for improved traffic safety, localizing road users in the 5 GHz band. Adv. Radio Sci..

[28] Nagai M., Nakaoka K., Doi Y. Pedestrian-to-vehicle Communication Access Method and Field Test Results. Proceedings of the International Symposium on Antennas and Propagation (ISAP).

[29] Ho P.F., Chen J.C. (2017). WiSafe: Wi-Fi and Pedestrian Collision and Avoidance System. IEEE Trans. Veh. Technol..

[30] Zadeh R.B., Ghatee M., Eftekhari H.R. (2018). Three-Phases Smartphone-Based Warning System to Protect Vulnerable Road Users Under Fuzzy Conditions. IEEE Trans. Intell. Trans. Syst..

[31] Lin C.H., Chen Y.T., Chen J.J., Shih W.C., Chen W.T. pSafety: A Collision Prevention System for Pedestrians Using Smartphone. Proceedings of the 84th Vehicular Technology Conference (VTC-Fall).

[32] Artail H., Khalifeh K., Yahfoufi M. Avoiding Car-Pedestrian Collisions Using a VANET to Cellular Communication Framework. Proceedings of the 13th International Wireless Communications and Mobile Computing Conference.

[33] Nakanishi Y., Yamaguchi R., Fujimoto K., Wada T., Okada H. A New Collision Judgment Algorithm for Pedestrian-Vehicular Collision Avoidance Support System (P-VCASS) in Advanced ITS. Proceedings of the 6th International Conference on Networking and Services.

[34] Li C.Y., Salinas G., Huang P.H., Tu G.H., Hsu G.H., Hsieh T.Y. V2PSense: Enabling Cellular-based V2P Collision Warning Service Through Mobile Sensing. Proceedings of the IEEE International Conference on Communications (ICC).

[35] Merdrignac P., Shagdar O., Nashashibi F. (2017). Fusion of Perception and V2P Communication Systems for the Safety of Vulnerable Road Users. IEEE Trans. Intell. Transp. Syst..

[36] Kwakkernaat M., Ophelders F., Vissers J., Willemsen D., Sukumar P. Cooperative Automated Emergency Braking For Improved Safety Beyond Sensor Line-Of-Sight And Field-Of-View. Proceedings of the FISITA 2014 World Automotive Congress.

[37] Thielen D., Lorenz T., Hannibal M., Koster F., Plaettner J. A Feasibility Study on a Cooperative Safety Application for Cyclists crossing Intersections. Proceedings of the 15th International IEEE Conference on Intelligent Transportation Systems.

[38] Hernandez-Jayo U., la Iglesia I.D., Perez J. (2015). V-Alert: Description and Validation of a Vulnerable Road User Alert System in the Framework of a Smart City. Sensors.

[39] Anaya J.J., Talavera E., Giménez D., Gómez N., Jiménez F., Naranjo J.E. Vulnerable Road Users Detection using V2X Communications. Proceedings of the 18th International Conference on Intelligent Transportation Systems.

[40] Tal I., Muntean G.M. V2X Communication-based Power Saving Strategy for Electric Bicycles. Proceedings of the Globecom Workshop—Vehicular Network Evolution.

[41] Lu Z.Q., Feng Y., Yang J., Zheng X.Q. An Improved Infrastructure Assisted V2P Protocol for VANETs. Proceedings of the 10th International Conference on Wireless Communications, Networking and Mobile Computing (WiCOM).

[42] TIMON (2017). Enhanced Real Time Services for an Optimised Multimodal Mobility Relying on Cooperative Networks and Open Data. https://www.timon-project.eu.

[43] Carpenter M.G., Feldmann M., Moury M.T., Skvarce J.R., Struck M., Zwicky T.D., Kiger S.M. (2013). Objective Tests and for and Forward-Looking Pedestrian and Crash Avoidance/Mitigation—Systems Annual Report.

[44] Swanson E.D., Yanagisawa M., Najm W., Foderaro F., Azeredo P. (2016). Crash Avoidance Needs and Countermeasure Profiles for Safety Applications Based on Light-Vehicle-to-Pedestrian Communications.

[45] Morris A., Hancox G., Martin O., Bell D., Johansson C., Rosander P., Scholliers J., Silla A. Critical accident scenarios for cyclists and how they can be addressed through ITS solutions. Proceedings of the International Cycling Safety Conference.

[46] USDOT (2015). NYC Connected Vehicle Project. https://www.cvp.nyc/cv-safety-apps.

[47] USDOT (2014). Connected Vehicles: Vehicle-to-Pedestrian Communication. http://www.its.dot.gov/factsheets/pdf/CV_V2Pcomms.pdf.

[48] ETSI 3GPP TS 22.185 V14.4.0 Service Requirements for V2X Services; Stage 1 (Release 14), 2018. www.3gpp.org/DynaReport/22185.htm/.

[49] Rostami A., Cheng B., Lu H., Kenney J.B., Gruteser M. Performance and Channel Load Evaluation for Contextual Pedestrian-to-Vehicle Transmissions. Proceedings of the First ACM International Workshop on Smart, Autonomous, and Connected Vehicular Systems and Services.

[50] OMNeT++ Network Simulation Framework. http://www.omnetpp.org/.

[51] Sommer C., German R., Dressler F. (2011). Bidirectionally Coupled Network and Road Traffic Simulation for Improved IVC Analysis. Trans. Mob. Comput..

[52] Krajzewicz D., Erdmann J., Behrisch M., Bieker L. (2012). Recent Development and Applications of SUMO—Simulation of Urban MObility. Int. J. Adv. Syst. Meas..

[53] Broadcom Corporation (2017). Superaccurate GPS Chips Coming to Smartphones in 2018. https://spectrum.ieee.org/tech-talk/semiconductors/design/superaccurate-gps-chips-coming-to-smartphones-in-2018.

